# Bridging a diagnostic Kawasaki disease classifier from a microarray platform to a qRT-PCR assay

**DOI:** 10.1038/s41390-022-02148-y

**Published:** 2022-06-22

**Authors:** Rowan Kuiper, Victoria J. Wright, Dominic Habgood-Coote, Chisato Shimizu, Daphne Huigh, Adriana H. Tremoulet, Danielle van Keulen, Clive J. Hoggart, Jesus Rodriguez-Manzano, Jethro A. Herberg, Myrsini Kaforou, Dennie Tempel, Jane C. Burns, Michael Levin

**Affiliations:** 1 SkylineDx, Rotterdam, The Netherlands; 2 Department of Infectious Disease, Imperial College London, London, UK; 3 Department of Pediatrics, Rady Children’s Hospital and University of California San Diego, La Jolla, CA USA; 4 Department of Genetics and Genomic Sciences, Icahn School of Medicine at Mount Sinai, New York City, NY USA

## Abstract

**Background:**

Kawasaki disease (KD) is a systemic vasculitis that mainly affects children under 5 years of age. Up to 30% of patients develop coronary artery abnormalities, which are reduced with early treatment. Timely diagnosis of KD is challenging but may become more straightforward with the recent discovery of a whole-blood host response classifier that discriminates KD patients from patients with other febrile conditions. Here, we bridged this microarray-based classifier to a clinically applicable quantitative reverse transcription-polymerase chain reaction (qRT-PCR) assay: the Kawasaki Disease Gene Expression Profiling (KiDs-GEP) classifier.

**Methods:**

We designed and optimized a qRT-PCR assay and applied it to a subset of samples previously used for the classifier discovery to reweight the original classifier.

**Results:**

The performance of the KiDs-GEP classifier was comparable to the original classifier with a cross-validated area under the ROC curve of 0.964 [95% CI: 0.924–1.00] vs 0.992 [95% CI: 0.978–1.00], respectively. Both classifiers demonstrated similar trends over various disease conditions, with the clearest distinction between individuals diagnosed with KD vs viral infections.

**Conclusion:**

We successfully bridged the microarray-based classifier into the KiDs-GEP classifier, a more rapid and more cost-efficient qRT-PCR assay, bringing a diagnostic test for KD closer to the hospital clinical laboratory.

**Impact:**

- A diagnostic test is needed for Kawasaki disease and is currently not available.
- We describe the development of a One-Step multiplex qRT-PCR assay and the subsequent modification (i.e., bridging) of the microarray-based host response classifier previously described by Wright et al.
- The bridged KiDs-GEP classifier performs well in discriminating Kawasaki disease patients from febrile controls.
- This host response clinical test for Kawasaki disease can be adapted to the hospital clinical laboratory.

## Introduction

Kawasaki disease (KD) is a systemic vasculitis of unknown etiology that is most prevalent in children under 5 years of age.^1–6^ KD patients are at risk of developing coronary artery abnormalities (CAA), which may result in thrombosis, stenosis, or occlusion, potentially leading to ischemic heart disease or even death.^7,8^ Timely treatment within the first week after fever onset with intravenous immunoglobulin (IVIG) reduces CAA incidence from 18–31% to 2–8%.^9–11^ However, delays in diagnosis and treatment are common. Recent studies show that on average, KD patients are treated on day 7 after disease onset, and that 2.9–16.8% of KD patients are treated after day 10.^12–17^ As each day of delayed treatment increases the chance of CAA development, KD patients treated after day 10 have a clear increased risk of developing CAA.^18,19^ The increased risk of giant coronary artery aneurysms, which more often lead to adverse events, is worrisome in these patients.^7^

Diagnosis of KD can be challenging as there is no diagnostic test available for KD. Therefore, the current diagnosis is based on clinical symptoms with supportive laboratory data. Following the American Heart Association (AHA) guidelines, a diagnosis of complete KD can be made if a patient has ≥5 days of fever in combination with ≥4 out of 5 principal clinical features: 1. bilateral conjunctival congestion, 2. redness of the lips, and oral mucosa, 3. polymorphous exanthema, 4. reddening of the palms and soles followed by membranous desquamation, and 5. acute non-purulent cervical lymphadenopathy.^7^ However, these clinical signs are not specific to KD and may also accompany other childhood diseases including measles, adenoviral infection, scarlet fever, and Stevens–Johnson syndrome. Furthermore, not all clinical signs may be present at the same moment in time and may have already resolved or have yet to manifest at the time the patient is examined by a physician.^20^ Finally, 20–30% of KD patients present with less than four principal KD criteria or symptoms that are uncommon in KD, i.e., incomplete KD.^7,21–23^ There is therefore a clear need for an improved diagnostic tool that can support an early diagnosis of KD.

In other clinical dilemmas where there is a lack of a quick and accurate diagnostic tool, such as the discrimination between bacterial and viral infections in febrile children, the host response has been suggested to provide a solution. Classifiers based on the host response gene expression containing only a few features are already able to distinguish bacterial from viral infections. These classifiers can be used together with traditional diagnostic methods to improve diagnostic accuracy and to support clinicians in differentiating bacterial from viral infections in febrile children.^24^ Recently, a host RNA classifier was described that can aid in the diagnosis of KD.^25^ This classifier discriminates between KD patients and patients with other febrile conditions, including patients with bacterial and viral infections, with high sensitivity and specificity. It was discovered using genome-wide RNA expression microarrays, which are very useful in research settings to quantify the expression of large numbers of genes in parallel, but are not suited for the acute clinical care setting. In clinical settings, a real-time quantitative reverse transcription PCR (real-time qRT-PCR)-based diagnostic tool would be more useful. It is a commonly used diagnostic technology that is simpler, targeted, and more cost-efficient than microarray, making it easier to adapt to a clinical setting.^26^ These characteristics are especially important for KD, for which a short turnaround time and affordability are vital to make a rapid diagnosis widely available for every child who is suspected of having KD.

Although microarray and qRT-PCR are both methods to measure gene expression, and multiple studies have shown concordant readouts for differentially expressed genes in acute KD patients, divergent results can occur due to inherent differences in the underlying experimental methodology and technology.^27–29^ Therefore, transferring a classifier to another technology platform while retaining its diagnostic accuracy and performance, requires effort to align readouts between platforms (i.e., bridging).

In this study, we bridge the 13-gene classifier discovered by Wright et al. from a microarray platform to a multiplex qRT-PCR assay.^25^ This comprises two steps: (1) developing the multiplex qRT-PCR assay and (2) modifying the original classifier such that it is applicable to the assay. The bridged classifier is termed the Kawasaki Disease Gene Expression Profiling (KiDs-GEP) classifier.

## Methods

### Patients

**Fig. 1:**
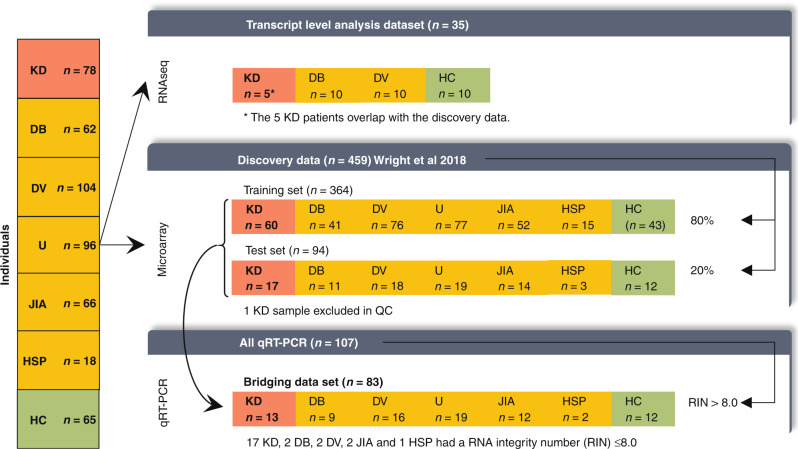
Study cohort overview. Overview of the data and samples used in this study and the techniques that were used to measure gene expression in the cohorts. KD Kawasaki disease, DB definite bacterial, DV definite viral, U infections of uncertain bacterial or viral etiology, JIA juvenile idiopathic arthritis, HSP Henoch–Schönlein purpura, HC healthy control. The microarray data and its subdivision into the training and test set were described by Wright et al., who excluded one microarray KD sample after a quality control analysis. The set of individuals measured by qRT-PCR is a subset of the individuals measured by microarray, for which sufficient RNA was available. The RNA-seq dataset is independent of the other sets, except for the five KD patients.

In this study, 489 individuals were included (Fig. 1) of which 459 were described previously, and 30 additional individuals were included from preexisting RNA sequencing (RNA-seq) studies.^25^ Subsets of these patients were analyzed within various datasets described below in the dataset section. Patients with KD, infectious conditions (including bacterial and viral infections), inflammatory conditions (including juvenile idiopathic arthritis and Henoch–Schönlein purpura), and healthy controls (children with no recent history of fever or immunization) were prospectively recruited at pediatric centers in the United Kingdom, Spain, the Netherlands, and the United States from March 1, 2009, to November 14, 2013, as previously described.^25^ Recruited KD patients represent a combination of patients seen directly in the emergency department and patients referred from regional centers. KD was diagnosed on the basis of the AHA criteria.^7^ All were enrolled within the first 7 days of illness, before initiation of IVIG. For the febrile controls, blood was drawn as soon as possible after presentation and before a clinical diagnosis was confirmed. Patients were assigned to diagnostic groups once the results of all investigations were available based on predefined criteria (Supplementary Fig. S1 and Supplementary methods). Children with comorbidities or taking medications likely to influence gene expression were excluded.

### RNA isolation and processing

Whole blood was collected (2.5 mL) in PAXgene blood RNA tubes (PreAnalytiX, Hombrechtikon, Switzerland), incubated for 2 h at ambient temperature, frozen at –20 °C within 24 h of collection, and stored at –80 °C. Total RNA was extracted using PAXgene blood RNA kits (PreAnalytiX) according to the manufacturer’s instructions.

### Datasets

#### Microarray, discovery dataset

For 459 individuals, gene expression profiles were determined on the HumanHT-12 v4 BeadChip (Illumina, San Diego, CA) by Imperial College London as described previously.^25^ The data are available on the Gene Expression Omnibus under accession number GSE73461. Details are provided as Supplementary material.

#### RNA-seq, transcript-level analysis dataset

Additional patient data with available RNA-seq data (strand specific 2 × 75 bp) was collected, consisting of 5 KD patients overlapping the microarray discovery data (same RNA aliquot), 10 bacterial, 10 viral, and 10 healthy controls. The shallowest sequenced sample in the analysis reached a depth of 32.4 M fragments that is sufficient to detect the most known splice junctions (Supplementary Fig. S2). More details are provided as Supplementary material.

#### qRT-PCR, bridging dataset

A One-Step multiplex qRT-PCR assay was run for a subset of individuals from the microarray discovery dataset (*n* = 107 of 459) for which sufficient total RNA was available. Prior to qRT-PCR, the total RNA integrity number (RIN) was assessed using the Bioanalyzer (Agilent, Santa Clara, CA). A total of 62.5 ng was used for the qRT-PCR of 15 targets (13 classifier genes + 2 reference genes described below), these targets were divided into 5 triplexes. The qRT-PCR was performed on the QuantStudio 5 Dx system (Applied Biosystems, Waltham, MA) using the Reliance One-Step Multiplex qRT-PCR Supermix (4×) from BioRad (Hercules, CA) with primers and probes described below. The following thermal cycling protocol was used: 10 min at 50 °C, 10 min at 95 °C and 40 cycles of 10 s at 95 °C and 30 s at 60 °C.

### qRT-PCR assay design

#### Identification of qRT-PCR reference genes

Using the microarray discovery dataset (batch corrected for effects between sites), a shortlist of potential reference genes was created (Supplementary Fig. S3 and Supplementary Table S1) based on the following properties. (1) Transcripts moderately to highly expressed (mean log_2_ expression between 11 and 15), (2) expression invariant to the disease condition (genes with univariate Limma *p* value < 0.05 for differential expression between disease conditions were excluded), and (3) minimal variance in expression between samples.^30^ Therefore, genes with more than three outliers as defined by the robust *Z* score method as 0.6745 $\frac{{x_i - {{{{{\mathrm{median}}}}}}(x)}}{{{{{{{{{\mathrm{median}}}}}}}}\;{{{{{{{\mathrm{absolute}}}}}}}}\;{{{{{{{\mathrm{deviate}}}}}}}}}} > 3.5$, were excluded. The remaining (*n* = 173) genes were sorted by increasing variance and assessed top-down for optimal primer/probe designs according to the specifications listed in the primer and probe design section below.

#### Primer and probe design

**Fig. 2:**
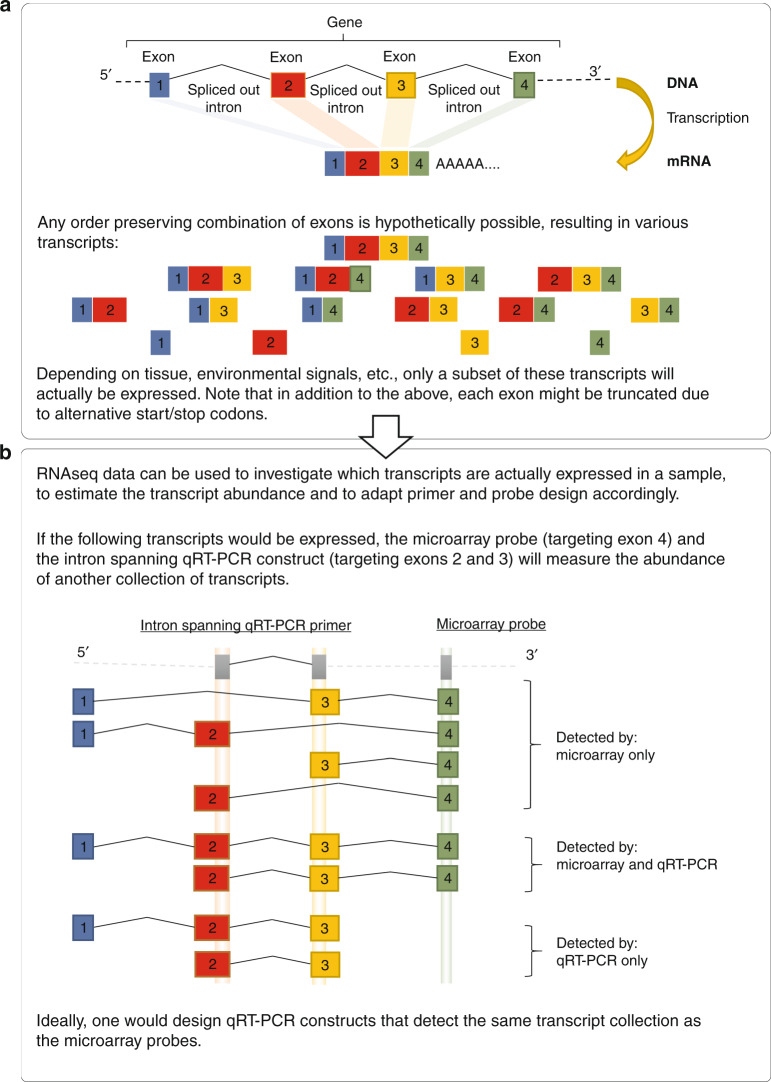
Transcript collections targeted by the qRT-PCR and microarray probes. **a** A gene can be represented as a collection of transcripts, characterized by alternative splicing, and alternative start and stop codons. **b** Not all transcripts will be expressed in the whole blood of individuals in the classifier’s intended population. Therefore, we observed the actual transcript expression in RNA sequencing data, and estimated the expression for both the microarray probe and the qRT-PCR construct, by aggregating the expressions of transcripts overlapping the probe.

qRT-PCR primers and probes (TaqMan, Applied Biosystems) were designed using Primer3 (Supplementary Table S2).^31^ The assay—measuring 15 targets in triplex with FAM, ABY and VIC labeled probes—was designed with the following rules considered: (1) reference genes should not be combined in a triplex, (2) target length of PCR product 75–140 bp, (3) in multi-exon transcripts, at least one primer should be intron spanning, (4) probes should not overlap with primers, (5) probes should have a melting temperature (Tm) of approximately 10 °C above the lowest primer Tm, and (6) the qRT-PCR primers should target similar transcript collections as the corresponding microarray probe (Fig. 2). This last point was assessed by a transcript-level analysis in the preexisting RNA-seq data. To minimize the creation of unwanted side products such as self-dimers and cross-dimers, specificity and target-specific binding were checked in silico by Primer-BLAST and Multiple Primer Analyzer (ThermoFisher Scientific, Waltham, MA), respectively.^32^ The performances of the probes were assessed by evaluating the amplification curves and expression profiles for each target in a singleplex qRT-PCR reaction. After triplex combinations were made, fluorescent dyes were assigned to specific probes with FAM labels assigned to probes targeting low abundance transcripts, VIC labels assigned to probes targeting high abundance transcripts, and ABY labels assigned to probes targeting moderate abundance transcripts.

#### Probe mapping

The HumanHT-12 v4 BeadChip probe sequences were obtained from the Gene Expression Omnibus platform annotation (Accession number: GPL10558). Together with the qRT-PCR constructs, they were aligned (Supplementary Table S3) to the GRCh38 reference genome by single-pass STAR (v2.7.8a) using the GENCODE release 38 gene model (evidence-based annotation in Ensembl 104) to generate the splice junction database.^33^

### Model building

#### Calculating qRT-PCR ΔCt

Within the qRT-PCR assay, target transcript levels were reported relative to two stably expressed reference genes as *ΔCt*_target_ = ½(*Ct*_ref1_ + *Ct*_ref2_)–*Ct*_target._^34^ For the current purpose, i.e., a robust outcome of the bridged classifier—the qRT-PCR amplification efficiencies were assumed not to vary within a gene, although they may vary between genes.

#### Reweighting a linear model

To bridge the 13-gene classifier from one gene expression representation to an alternative representation (e.g., microarray to qRT-PCR), we required both methods to be performed on the same patient material (i.e., paired samples), and assumed that expression measurements between both platforms are linearly related. A mathematical description of the method is detailed in the Supplementary methods. Briefly, a multiple linear regression was performed to express the microarray log_2_ intensity values in terms of all qRT-PCR ΔCt values to be included in the model. The reweighted model was obtained by substituting the found regression coefficients into the original model.

#### Cross-validation

The performance of the bridged model was assessed by calculating risk scores in a leave-one-out cross-validation, which was compared against the predictions of the original model.^35^ Within each cross-validation loop, one sample was left out. Based on the remaining samples, inner fold models were built by the reweighting procedure. Using the inner fold models that were independent of the left-out sample, scores were calculated for the left-out sample. Subsequently, in the next fold, another sample was removed, until all were drawn once. At that point, the predicted scores were compared with the scores obtained by the original model.

#### Statistical analysis

All analyses were done in R (v4.0.3).^36^ Linear regression was performed with the “lm” function. Principal components were determined using the “prcomp” function. Wilcoxon rank-sum and Fisher exact statistics were calculated using the “wilcox.test” and “fisher.test” functions, respectively. Receiver operator characteristics (ROC) and related metrics such as area under the curve (AUC) with 95% confidence interval (95% CI), and Youden J statistics were calculated by the pROC package (v1.17.0.1).^37^

## Results

### Identification of potential reference genes

**Fig. 3:**
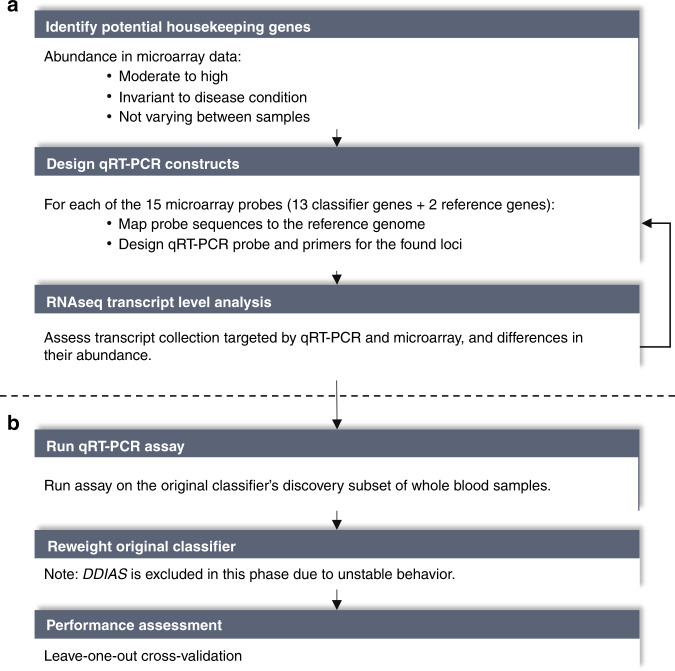
Methodological flowchart of bridging steps. **a** Design of the one-step multiplex qRT-PCR assay, and **b** adjustment of classifier weights by comparison of readouts of paired samples (microarray vs qRT-PCR), and assessment of the performance of the bridged model.

An overview of the entire bridging process is shown in Fig. 3, detailing the design of a new one-step multiplex qRT-PCR assay, and subsequent modification of the original classifier into the KiDs-GEP classifier. Based on the microarray discovery data, *AURKAIP1* and *SSU72* were the highest-ranked potential reference genes meeting our predefined criteria for the design of qRT-PCR primers and probes and were therefore selected as reference genes for the qRT-PCR assay.

### Probe mapping

For each of the 15 microarray probes (13 classifier genes + 2 reference genes), multiplex qRT-PCR assay primers and probes were designed (Supplementary Table S2). All microarray probe sequences were mapped to the reference genome (Supplementary Table S3). The *ILMN_1898691* probe targets a locus on the positive strand of chromosome 3 without an associated gene in GENCODE. There is however a predicted transcript (GenBank Accession: XR_002959502.1) for *TIGIT* that overlaps the locus. As this transcript was expressed in the RNA-seq data, we designed the corresponding qRT-PCR construct, spanning the nearest intron within *TIGIT*.

### Transcript-level analysis

A gene can be represented as a collection of transcripts, characterized by alternative splicing, and alternative start and stop codons (Fig. 2). It is important that the qRT-PCR primers and probes target the same transcript collection as the microarray probes because these transcripts may be differentially expressed. Therefore, the overlap with known transcripts for each of the 15 genes of interest was determined in silico for both the microarray and qRT-PCR probes (Supplementary Table S4). Although in general there was a large overlap in the set of transcripts targeted by both platforms, for most genes there were transcripts that were targeted by only one of the platforms.

**Fig. 4:**
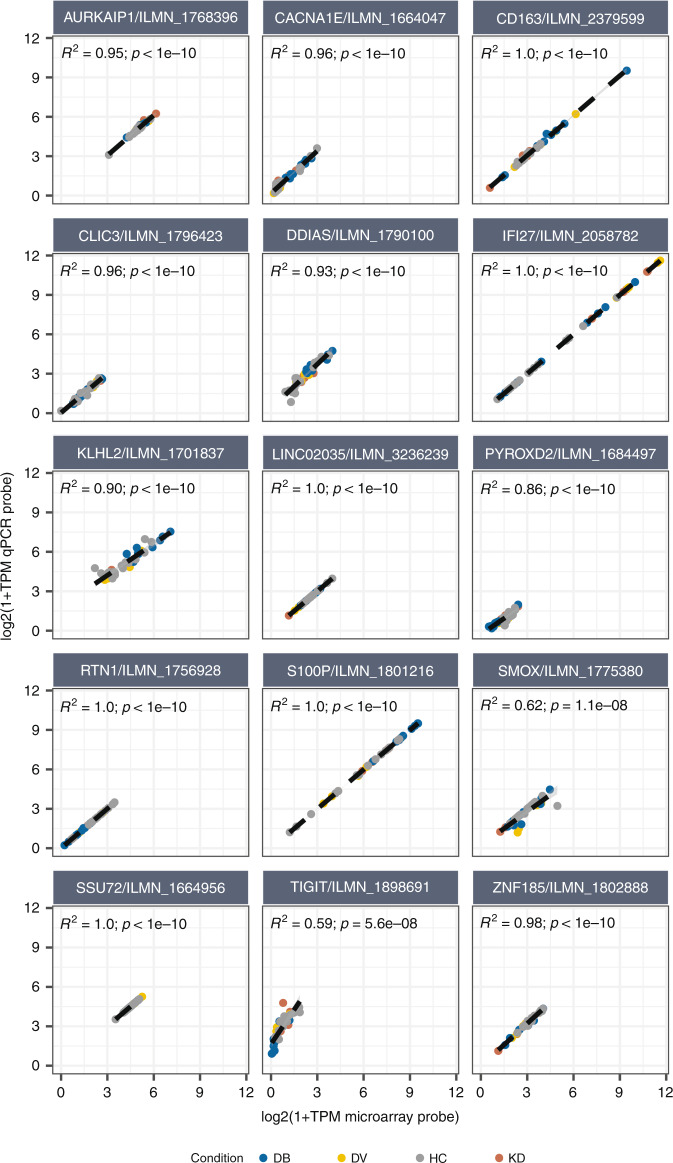
Relation between RNA-seq-based estimation of qRT-PCR vs microarray probes abundance levels. Abundance levels are based on the aggregated transcripts that overlap the qRT-PCR (vertical axis) and microarray probes (horizontal axis), considering similar strand orientations. Colors indicate condition: Kawasaki disease (red), definite bacterial (blue), definite viral (yellow), and healthy control (gray). TPM transcripts per million RNA-seq reads.

Not all transcripts will be expressed or detected in the whole blood of individuals in the classifier’s intended population. Therefore, we observed the actual transcript abundance in the RNA-seq data, and estimated the abundance for both the microarray probe and the qRT-PCR construct, by aggregating the level of abundance of transcripts overlapping the probe (Fig. 4). For 12 genes, the correlation between the estimated microarray and qRT-PCR transcript abundance was high (*R*^2^ ≥ 0.90), suggesting that measuring transcript abundance with the qRT-PCR probes instead of the microarray probes had no major effect on the quantification of transcripts in the intended population. The lowest correlations were seen for *PYROXD2* (*R*^2^ = 0.86; *p* < 1 × 10^–10^), *SMOX* (*R*^2^ = 0.62; *p* = 1.1 × 10^–8^) and *TIGIT* (*R*^2^ = 0.59; *p* = 5.6 × 10^–8^). Although the correlation observed for *TIGIT* was lower, and thus was affected by using the qRT-PCR probes instead of the microarray probes, there was a clear non-random correlation indicating that the microarray probe indeed targets *TIGIT*.

### Correlation microarray and qRT-PCR data

To bridge the 13-gene signature from microarray to qRT-PCR, we employed gene expression measurements for 107 individuals from both the microarray and qRT-PCR platforms (Fig. 1). To ensure the accuracy of quantification, only samples with a RIN > 8.0—commonly considered high-quality RNA—were included in subsequent analyses (*n* = 83), unless mentioned otherwise.^38^

**Fig. 5:**
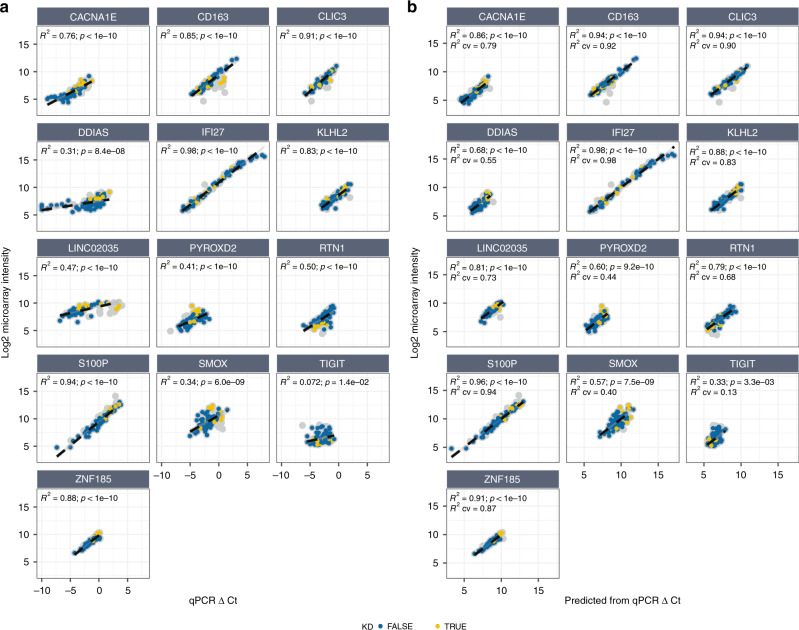
Pre- and post-modeling of the qRT-PCR expressions. **a** Pre-modeling, showing the observed microarray log2 intensity values versus the qRT-PCR ΔCt values, and **b** post-modeling, showing the predicted log2 intensity values based on 12 qRT-PCR probes. Colors indicate KD (yellow), non-KD (blue) or excluded due to RIN ≤ 8 (gray). The raw measurements show linear associations for all of the 13 genes. Post-modeling correlations are higher and reduce the undesirable effects seen in some genes. The full regression models are shown in Supplementary Table S6A–M. For the purpose of visualization only, undetectable qRT-PCR ΔCt values in *DDIAS* have been set to –10.

Clinical characteristics, demographics, and microarray results for the 15 genes did not differ between the bridging dataset and the discovery training set used for building the original model (Table 1 and Supplementary Table S5A–D), suggesting a representative sampling of the discovery set. By comparing the log_2_ normalized microarray intensity values with the ΔCt qRT-PCR values, positively correlated linear relations were observed for all 13 genes (Fig. 5a). There was a level of co-regulation between genes as suggested by the observed correlations (Supplementary Fig. S4). Although correlations between platforms were mostly highest between matching probes (e.g., the correlation for *IFI27* between microarray and qRT-PCR), this was not true for *DDIAS, LINC02035, TIGIT*, and *SMOX*, suggesting platform specific-differences for these probes. For example, the correlation for *DDIAS* between microarray and qRT-PCR was lower than the correlation between *DDIAS* on microarray vs *KLHL2* on qRT-PCR.

**Table 1:** Patient characteristics.

A	Discovery data										
			KD	DB	DV	U	JIA	HSP	HC		
Total		*n*	77	52	94	96	66	18	55		
Age	[month]	Med (IQR)	26 (16–45)	22 (9–46)	14 (2–40)	27 (7–71)	160 (130–183)	55 (42–80)	38 (20–76)		
Sex		% male/female	55/45	42/58	70/30	65/35	38/62	50/50	53/47		
Ethnicity		% A/B/C/H/O^e^	16/ 4/ 26/ 33/ 21	10/4/73/0/12	6/11/53/16/14	21/14/55/2/7	0/5/80/0/15	0/0/25/25/50	11/11/46/0/32		
Illness day	[day]	Median (IQR)	5 (4–6)	6 (3–10)	5 (4–7)	6 (4–9)		4 (2–6)			
*Z* score		Med (IQR)	1.9 (1.5–2.5)								
CA status		% N/D/A^f^	57/10/32								
WBC	[×10^3^/µL]	Median (IQR)	14.0 (10.4–18.1)	12.6 (7.7–19.3)	8.5 (6.1–11.9)	8.4 (6.5–14.6)	6.2 (5.2–6.8)	11.7 (9.7–14.1)	7.2 (6.4–9.8)		
Platelets	[×10^3^/µL]	Median (IQR)	355 (306–448)	181 (134–279)	267 (202–353)	270 (204–338)		340 (264–457)	349 (280–414)		
Neutrophils	[×10^3^/µL]	Median (IQR)	9.5 (7.1–12.2)	6.7 (3.9–15.0)	4.2 (2.8–7.2)	4.9 (3.2–10.1)	3.0 (2.3–3.7)	6.5 (4.2– 8.6)	2.9 (2.5–4.6)		
ESR	[mm/h]	Median (IQR)	58.0 (34.0–78.0)		35.0 (31.0–50.0)	44.0 (41.0–45.0)	5.0 (2.0–11.0)	0.0 (0.0–0.0)			
CRP	[mg/L]	Median (IQR)	118.0 (48.0–192.0)	126.5 (67.8–218.8)	17.2 (5.0–35.0)	80.0 (37.0–145.0)	1.0 (0.3–3.0)	22.0 (7.8–24.0)	1.0 (1.0–1.0)		

B	Bridging data									C	Both
			KD	DB	DV	U	JIA	HSP	HC		*P*
Total		*n*	13	9	16	19	12	2	12		0.99^a^
Age	[month]	Median (IQR)	29 (24–45)	19 (9–34)	16 (1–35)	27 (8–52)	137 (115–140)	85 (75–95)	30 (16–55)		0.42^c^
Sex		% male/female	46/54	33/67	69/31	74/26	25/75	50/50	75/25		0.97^d^
Ethnicity		% A/B/C/H/O^e^	0/0/54/23/23	0/12/88/0/0	7/7/53/13/20	22/22/50/0/6	0/8/67/0/25	0/0/0/0/100	12/0/62/0/25		0.76^d^
Illness day	[day]	Median (IQR)	6 (5–7)	5 (4–8)	5 (4–7)	6 (5–10)		2 (1–2)			0.63^c^
*Z* score		Median (IQR)	1.9 (1.5–2.5)								1.0^b^
CA status		% N/D/A^f^	62/8/31								1.0^a^
WBC	[×10^3^/µL]	Median (IQR)	11.9 (10.5–14.4)	10.5 (7.7–13.3)	8.9 (7.2–10.3)	8.4 (6.7–13.8)	5.9 (5.4–6.9)	11.0 (10.7–11.3)	6.7 (5.9–8.8)		0.37^c^
Platelets	[×10^3^/µL]	Median (IQR)	332 (322–415)	145 (105–193)	252 (238–444)	268 (159–352)		292 (284–299)	352 (320–379)		0.52^c^
Neutrophil	[×10^3^/µL]	Median (IQR)	9.4 (8.4–9.8)	5.7 (5.3–10.2)	4.0 (2.6–5.6)	4.9 (3.5–9.6)	2.9 (2.0–3.5)	8.0 (8.0–8.0)	3.0 (2.7–3.5)		0.47^c^
ESR	[mm/h]	Median (IQR)	56.0 (37.0–65.0)		43.0 (39.0–47.0)		6.5 (5.0–8.8)	12.0 (12.0–12.0)			0.76^c^
CRP	[mg/L]	Median (IQR)	80.0 (23.0–194.0)	169.0 (81.0–183.0)	14.0 (7.0–44.1)	66.0 (21.0–150.5)	0.8 (0.1–2.2)	39.0 (31.0–47.0)			0.48^c^

**A** Discovery data, **B** bridging data, and **C** comparison between discovery and the bridging data, per characteristic. Conditions without known values are left blank.

*KD* Kawasaki disease, *DB* definite bacterial, *DV* definite viral, *U* infections of uncertain bacterial or viral etiology, *JIA* juvenile idiopathic arthritis, *HSP* Henoch–Schönlein purpura, *HC* healthy control, *CA status* coronary artery status, *WBC* white blood cells, *ESR* erythrocyte sedimentation rate, *CRP* C-reactive protein.

^a^Fisher exact test, ^b^Wilcoxon rank-sum, ^c^van Elteren test, ^d^Cochran–Mantel–Haenszel test, ^e^Ethnicity: A = Asian, B = Black, C = Caucasian, H = Hispanic, O = Others, ^f^CA status: N = Normal, D = Dilated, A = Aneurysm. See Supplementary Table S5A–D for a comparison of expression values between datasets.

Genes affected by alternatively targeted transcripts in the RNA-seq analysis were indeed among the genes with the poorest correlations in this microarray vs qRT-PCR dataset. There also appeared to be a relationship with gene expression level; lower abundance genes tended to have lower correlations (Supplementary Fig. S5). For genes that were expressed with great variability among samples, the correlations were highest, as was most prominently seen for *IFI27* and *S100P* having differences of >9 between the highest and lowest log_2_ microarray intensities, of which 98% (*p* < 1 × 10^–10^) and 94% (*p* < 1 × 10^–10^) of their variances were captured by the qRT-PCR assay respectively. Other genes with less dynamic expression ranges and no evidence of targeting alternative transcripts, generally tended to show a good correlation between platforms. In contrast, there was a low correlation for *TIGIT*—the qRT-PCR assay only explained 7% (*p* = 0.01) of the variance in the microarray. Other relatively low correlations between platforms (*R*^2^ < 0.5) were observed for *DDIAS* (*R*^2^ = 0.30; *p* = 8.4 × 10^–8^), *SMOX* (*R*^2^ = 0.34; *p* = 6.0 × 10^–9^), *PYROXD2* (*R*^2^ = 0.41; *p* < 1 × 10^–10^), and *LINC02035* (*R*^2^ = 0.47; *p* < 1 × 10^–10^).

### Bridging

**Fig. 6:**
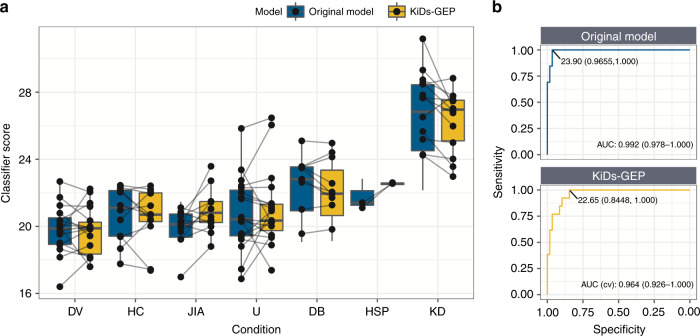
Comparison of performance in the bridging subset between the original 13-gene classifier (blue) and the bridged KiDs-GEP classifier (yellow). **a** The boxplot shows classifier scores per model for each condition (KD Kawasaki disease, DB definite bacterial, DV definite viral, U infections of uncertain bacterial or viral etiology, JIA juvenile idiopathic arthritis, HSP Henoch–Schönlein purpura, HC healthy control). Paired samples are connected by a line. **b** The ROC curve is shown for the original model (top) and the cross-validated curve for the KiDs-GEP model (bottom). The healthy controls and samples with RIN ≤ 8.0 were excluded from this analysis. The Youden coordinate (threshold, specificity and sensitivity), and AUC with 95% CI are given in text labels.

We bridged the microarray-derived 13-gene classifier to render it applicable to the qRT-PCR assay by reweighting the original model coefficients by means of multiple linear regression (Table 2A).^25^ As the *DDIAS* qRT-PCR probe showed unstable behavior resulting in undeterminable Ct values, this qRT-PCR probe was not included as an explanatory term in the model. After expressing the 13 microarray probes in terms of 12 qRT-PCR ΔCts, the correlations increased, especially for those genes that had the lowest correlations prior to the modeling (Fig. 5 and Supplementary Table S6A–M). For example, even though *DDIAS* was not included as a term in the reweighted model, its co-regulation with other genes in the model caused the information to be retained, and even improved the predicted microarray expression relative to the pre-modeling situation (*R*^2^ = 0.31 vs *R*^2^-cv = 0.55). A similar effect was seen for *LINC02035*, resulting in an improved correlation (*R*^2^ = 0.47 vs *R*^2^-cv = 0.73). The KiDs-GEP model was obtained by substituting the qRT-PCR-based modeled expressions into the original 13-gene model (Table 2B, C). The cross-validated AUC and 95% CI were 0.964 [0.924–1.00], which was in line with the observed AUC of 0.992 [0.978–1.00] for the original model (Fig. 6). Both classifiers demonstrated similar trends over the various disease conditions, with a clearest classifier score distinction between individuals diagnosed with KD and viral infections, showing interquartile ranges of [KD: 25.0–27.5] and [viral: 18.7–20.5] for the KiDs-GEP classifier versus [KD: 25.7–28.6] and [viral: 19.0–20.5] for the original model. In comparison, Individuals diagnosed with bacterial infections had a classifier score with interquartile ranges of [bacterial: 21.7–24.1] for the KiDs-GEP model and [bacterial: 22.6–23.6] for the original model.

**Table 2:** Three alternative representations of the KD classifier models.

A	Original	B	KiDs-GEP (mean/VAR)			C	KiDs-GEP
Illumina probe	Weight	qRT-PCR probe	Weight	Mean	SD	qRT-PCR probe	Weight
INTERCEPT	–	INTERCEPT	21.720	–	–	INTERCEPT	23.972
ILMN_1664047	0.955	CACNA1E	0.746	–4.763	1.751	CACNA1E	0.426
ILMN_1790100	0.844	DDIAS	–	–	–	DDIAS	–
ILMN_1701837	0.789	KLHL2	1.227	–0.865	1.299	KLHL2	0.945
ILMN_1684497	0.727	PYROXD2	0.347	–4.385	1.149	PYROXD2	0.302
ILMN_1775380	0.675	SMOX	1.061	–1.885	1.182	SMOX	0.898
ILMN_1802888	0.646	ZNF185	0.044	–1.185	0.863	ZNF185	0.051
ILMN_3236239	0.561	LINC02035	0.776	–3.382	2.029	LINC02035	0.382
ILMN_1796423	0.464	CLIC3	0.161	–2.690	1.244	CLIC3	0.129
ILMN_1801216	–0.405	S100P	0.379	0.591	2.114	S100P	0.179
ILMN_2058782	–0.426	IFI27	–1.138	–0.309	3.819	IFI27	–0.298
ILMN_1898691	–0.599	TIGIT	–0.196	–2.959	1.014	TIGIT	–0.193
ILMN_2379599	–0.638	CD163	–1.870	–1.791	1.497	CD163	–1.249
ILMN_1756928	–0.69	RTN1	–0.905	–2.643	1.037	RTN1	–0.873

**A** The original model that is applicable to the log_2_ intensity values of the Illumina HumanHT-12 v.4 Expression BeadChip, **B** the KiDs-GEP model applied to the mean/variance normalized ΔCt values of the qRT-PCR assay, and **C** the same KiDs-GEP model applied to the raw ΔCt values of the qRT-PCR assay. See Supplementary Table S5B–D for means and variances as observed per condition in the microarray and qRT-PCR data.

When applying the original 13-gene classifier or the KiDs-GEP classifier to samples with RIN ≤ 8.0 or non-quantifiable RIN, no evidence was found for a bias in classifier score related to RIN values (Supplementary Fig. S6). The average classifier score for the 13 KD samples with RIN values ≤8.0 did not differ significantly from the 14 KD samples with RIN > 8 in a Wilcoxon rank-sum exact test (KiDs-GEP: *p* = 0.58 and original model: *p* = 0.35).

## Discussion

A molecular test for diagnosing KD is currently not available, but would be a valuable tool to aid clinicians in the timely diagnosis of KD. The original description of the 13-gene classifier was an important first step, but required translation to the clinical acute care setting. In the current study, we describe the development of a qRT-PCR assay and subsequent bridging of the microarray-based 13-gene classifier into a clinically applicable qRT-PCR test. We also provide robust justification for two reference genes that could be generally adopted for whole-blood gene expression studies in KD research.

Prior to modeling, varying degrees of correlation were observed between microarray and qRT-PCR expression levels (*R*^2^ ranging from 7% to 98%). *DDIAS* in particular showed unstable behavior resulting in undeterminable Ct values. Therefore, we concluded that the designed *DDIAS* qRT-PCR construct was not sufficiently reliable for the assay, and it was not taken into account for the KiDs-GEP.

Several intrinsic differences between the platforms led to disparities in the quantitation of transcript abundance. First, many of the largest discrepancies were observed in the lowest abundant transcripts, indicating that depending on expression level, the sensitivity and specificity are affected differently in both platforms, perhaps due to altered amplification efficiency or cross-hybridization level. A second likely cause of differences in measured transcript abundance between the two different platforms may be the different strategies to reduce genomic DNA contamination. In the microarray, an oligo-dT primer is used to prime the poly-A tail to amplify all mRNAs simultaneously, hence microarray probes are often located distant from introns, at the far 3’-end. In qRT-PCR, sensitivity to genomic contamination is reduced by using an intron-spanning primer. This constraint eliminates the possibility to position a qRT-PCR construct at the same locus as its cognate microarray probe. Even though probes are targeting the same gene, they may be targeting different transcripts. A different challenge was observed with *LINC02035*, for which it was not possible to design an intron-spanning primer as the gene has no introns. Therefore, especially for this gene, the qRT-PCR assay might amplify genomic DNA. Indeed, we observed higher transcript abundance by qRT-PCR in a subset of samples for this particular construct, underlining the importance of incorporating methods to reduce amplification of genomic sequences. Despite earlier reports stating that probes for microarray and for RT-PCR should be close together in order to have highly correlated quantitation of transcripts, the intron nearest the 3’-end may not always be optimal due to alternative splicing.^39^ Therefore, analyzing RNA-seq data can be helpful to guide qRT primer design.

The poorest correlation between platforms was observed for *TIGIT*. The microarray *TIGIT* probe is unusual in several respects. Of all the 13 genes, *TIGIT* had the lowest expression level (Supplementary Fig. S5), was outside the common gene annotation for *TIGIT*, and another gene, *ZBTB20*, is expressed on the opposite strand. The possibility that the microarray probe actually targets both strands was excluded after consultation with the manufacturer. Therefore, it is likely that the low correlation between the two platforms was caused by low *TIGIT* expression levels. Despite the low correlation, the effect on the KiDs-GEP classifier was minimal, as the mean/variance normalized weight corresponding to *TIGIT* was among the smallest.

A number of samples had a RIN ≤ 8.0 or non-quantifiable RIN at the time of running the qRT-PCR assay. Many of those samples demonstrated higher *LINC02035* expression levels on qRT-PCR than on microarray. As discussed above, this could be due to genomic contamination, which is supported by visual inspection of the Bioanalyzer electropherograms (Supplementary Fig. S7). Interestingly, the effect of the suspected genomic contamination was less pronounced in the modeled data, because the model weights were redistributed over other genes that were less susceptible to DNA contamination (Supplementary Table S6G).

We successfully reweighted the classifier to the qRT-PCR data. This KiDs-GEP classifier distinguishes KD from other febrile conditions with a performance comparable to the original report. The classifier’s best performance was in the separation of KD patients from patients with a known viral infection. Children with viral infections form a large group who are often difficult to distinguish from KD based on clinical signs. Another major group that is difficult to distinguish from KD is patients with bacterial infections due to the overlap in inflammatory features between bacterial infection and KD. Therefore, a clinically useful classifier needs to distinguish KD from both bacterial and viral illness, for which the KiDs-GEP shows good performance.

Other host response classifiers for KD that have been described include: a 25-gene classifier that discriminates KD from adenovirus infection, a 10-gene classifier that discriminates the KD from group A streptococcus infection, and a 332-gene classifier that discriminates KD from both viral and bacterial infections.^40,41^ All were identified using microarray and have, to the best of our knowledge, not yet been bridged to a qRT-PCR assay or any similar platform.

The development of the KiDs-GEP assay is a significant step towards the development of a diagnostic test for KD. However, before such a test can be introduced in the clinic, the performance of the KiDs-GEP must be clinically and analytically validated. The primary aim of the clinical validation would be to confirm the performance in patients similar to those currently tested. Other clinical questions to be addressed include the performance of the classifier in subsets of KD patients, including those with CAA, those with incomplete clinical criteria, and infants. A study by Jaggi et al. showed that the gene expression profile of incomplete KD patients is almost identical to complete KD patients. It will be interesting to investigate if this is the case for the KiDs-GEP prediction as well.^40^ The performance of the classifier in discriminating KD from the multisystem inflammatory syndrome in children should also be tested.^42^ For analytical validity, key factors determining the stability of the assay must be investigated, including the stability of amplification efficiency as well as the robustness of the KiDs-GEP against samples with lower RIN values.

A limitation encountered in the current study was the available sample size. The original 13-gene classifier was built to predict the dichotomized class labels KD vs. non-KD in RNA from 60 KD patients, 261 febrile, and 43 healthy controls. After the selection of patients with sufficient RNA and a RIN > 8.0 for the bridging, 83 patients remained of which 13 were KD patients. This number is too small to perform subanalyses: incomplete vs. complete, CAA vs. no CAA, and IVIG responders vs. IVIG non-responders. A validation study will be required to gain insight into the role and performance of KiDs-GEP in these subgroups. In addition, given the sample size, remodeling a new classifier from scratch would suffer from reduced power relative to the original model, with a high probability of severe over-fitting of the data, and in the end would likely result in a classifier dissimilar from the one intended to be bridged. Therefore, instead of remodeling, we reweighted the original model coefficients by means of multiple linear regression. Modeling against the continuous expression instead of the dichotomized disease labels increases power.^43^ In this approach, the gene associations with outcome, and their corresponding original model weights are considered to be established, and the relation between the two representations was required to be linear.

In conclusion, we successfully bridged the microarray-based 13-gene classifier into the KiDs-GEP classifier, a faster and less costly 14-gene (i.e., 12 disease related + 2 reference genes) qRT-PCR assay, which brings this classifier based on host response one step closer to implementation in the clinical hospital laboratory.

## Supplementary information

20211215_Bridging paper_supplemental Tables

SupplementalDocument

## Data Availability

The data analyzed during the current study are available in the Gene Expression Omnibus under accession number GSE73461, included in this article and its supplementary information, or available upon request.
