# Supplementary material for: Bridging a diagnostic Kawasaki disease classifier from a microarray platform to a qRT-PCR assay
Source: Pediatr Res. 2022 Jun 22;93(3):559–69. doi: 10.1038/s41390-022-02148-y (PMC9988687; doi:10.1038/s41390-022-02148-y)
Supplement: Supplementary file 2 — SupplementalDocument [file 41390_2022_2148_MOESM2_ESM.pdf]

Supplementary Methods and Figures

Disclaimer: The methods provided in this supplementary data overlap significantly with Wright *et al*. but are included here to ensure the completeness of the manuscript.[1]

Materials and methods

Pathogen diagnosis

Viral diagnostics were undertaken on nasopharyngeal aspirates using immunofluorescence (RSV, adenovirus, parainfluenza virus, influenza A+B) and nested PCR (RSV, adenovirus, parainfluenza 1-4, influenza A+B, bocavirus, metapneumovirus, rhinovirus/enterovirus). Bacterial cultures included blood, cerebrospinal fluid (CSF), urine and tissue sites. Pneumococcal antigen was measured in blood and urine, and bacterial DNA was detected by meningococcal and pneumococcal PCR.

Diagnostic process in febrile controls

Patients had a diagnostic work-up as directed by the clinical team, including blood count, blood chemistry, C reactive protein (CRP), blood urine and throat swab cultures; CSF analysis and chest radiographs were performed where appropriate. Multiplex PCR was used to detect common respiratory viruses in nasopharyngeal aspirates or throat swabs, and common viruses in blood. Once the results of all investigations were available, patients were assigned to diagnostic groups using predefined criteria (Figure S1), as follows:

Bacterial infection: Patients assigned to the bacterial pathogen group had a bacterial pathogen (gram-positive coccus or gram-negative bacillus) identified by culture or by molecular techniques in a sample from a sterile site (blood, CSF, pleural space, joint, urine), and a clinical syndrome in keeping with the identified bacterial species. This group included patients with and without viral co-infection. Children diagnosed with other bacterial infections (for instance

mycoplasma, pertussis, mycobacteria) were not included in this group. No threshold for inflammatory markers was set for this group, as identification of bacteria in a sterile-site sample was taken as conclusive evidence for a confirmed bacterial infection.

Viral infection: Patients in the viral infection group had an identified virus, a clinical syndrome in keeping with viral infection, and no microbiological or clinical features of bacterial disease. In order to avoid inclusion of children with occult bacterial infection in the viral group, children with raised inflammatory markers were excluded. A maximum threshold was set at CRP of 60mg/L, and neutrophil count of 12 x 10^9 /L.

Uncertain bacterial or viral infection: When children with an acute febrile illness and features of infection could not be assigned confidently to either the viral or bacterial group, they were labelled as 'Uncertain Bacterial or Viral'. Children in this group had inconclusive features of bacterial or viral infection, negative microbiological findings or absent virological investigations, a syndrome inconsistent with their microbiological findings, inflammatory markers inconsistent with other clinical features of their illness, or insufficient clinical data for confident coding in another group. Patients in this group did not have bacterial infection detected at a sterile site, and some patients did have detectable virus.

Other inflammatory syndromes: a) Henoch–Schönlein purpura (HSP) was diagnosed in children presenting with palpable purpura, typically over the buttocks and extensor surfaces in association with abdominal pain, arthralgia or renal abnormalities (hematuria and proteinuria); b) Juvenile idiopathic arthritis (JIA) was defined according to International League of Associations for Rheumatology. Patients with JIA included i) treatment naïve and ii) active-exacerbation/smoldering.

Generating microarray data

As reported by Wright *et al*., total RNA yield was assessed using an Agilent 2100 Bioanalyzer and a NanoDrop 1000 spectrophotometer. After quantification and quality control, biotin-labelled cRNA was prepared using Illumina TotalPrep RNA Amplification kits (Applied Biosystems) from 500ng RNA. Labelled cRNA was hybridized overnight to HumanHT-12 v4 BeadChip (Illumina). After washing, blocking and staining, the arrays were scanned using an Illumina BeadArray Reader according to the manufacturer's instructions. Using Genome Studio software, the microarray images were inspected for artifacts and QC parameters were assessed. Background subtraction and robust spline normalization (RSN) were applied to the raw expression data using the R package lumi. In subsequent data quality control analyses, one KD samples was found to be an outlier in a principal component analysis and was excluded. The resulting intensity values for 47323 probes have been published in the Gene Expression Omnibus under accession number GSE73461.

Generating RNAseq data.

After an additional DNAse treatment step (Invitrogen), library preparation on whole blood total RNA was performed using the Illumina's TruSeq®RNA Sample Preparation Kit (2x75bp), ribosomal and globin RNA depletion was performed using the Illumina Ribo-Zero Gold kit and HiSeq 4000 at The Wellcome Centre for Human Genetics. Quality and adapter trimming of fastq files was performed by Cutadapt (v3.4) using default settings.[2] Resulting fastq files were aligned to the GRCh38 reference genome with double pass STAR (v2.7.8a) using the Gencode release 38 gene model (plus a manually added XR_002959502.1 transcript) to generate the splice junction database.[3] Using samtools/htslib (v1.10), the resulting BAM files were sorted and filtered to contain only mapping quality scores ≥200.[4] Downstream analyses on BAM files were performed using the R Bioconductor GenomicAlignments package.[5,6] Transcriptome mapping - generated from the intersection of the gene model with the genome

- was performed by Salmon (v1.4.0) using the complete genome as decoy.[7] A splice junction saturation analysis was performed using RseQC v4.0.0.[8]

Reweighting procedure

In the following sections, vectors are considered to be column vectors. Columns in matrices are denoted by a single index. E.g. $\boldsymbol{x_j}$ corresponds to the jth column in matrix $\boldsymbol{X}$. To deal with intercepts, a column vector of ones is sometimes added to matrices as column index 0, which is notated with a dot, e.g. $\dot{\boldsymbol{X}} = [\mathbf{1}, \boldsymbol{X}]$. Otherwise, matrix and vector indices start at 1. Similarly, vectors or matrices of regression coefficients might contain an intercept at index 0, notated as $\dot{\boldsymbol{\beta}} = [\beta_0, \boldsymbol{\beta}]$, or $\dot{\boldsymbol{B}} = [\boldsymbol{\beta}_0, \boldsymbol{B}]^T$respectively.

Reweighting a linear model

To bridge a model from one gene expression representation to an alternative representation (e.g. microarray to qRT-PCR), we require both methods to be performed within the same patients, and that the alternative representation is linearly related to the original representation. Let the original model be $\boldsymbol{y} = \dot{\boldsymbol{X}}\dot{\boldsymbol{\alpha}}$, expressing the response vector $\boldsymbol{y}$ as a linear function of expression matrix $\dot{\boldsymbol{X}}$ weighted by vector $\dot{\boldsymbol{\alpha}}$.

Gene expression measurements $x_{ij} \in \mathbf{X}$ and $z_{ik} \in \boldsymbol{Z}$ are obtained from the same patients indexed by $i = 1, \dots, n$, but with alternative (possibly disjoint) representations of features indexed by $j = 1, \dots,$ n, and $k = 1, \dots,$ s, respectively.

The multiple linear regression link model $\boldsymbol{X} = \dot{\boldsymbol{Z}}\dot{\hat{\boldsymbol{\Phi}}} + \boldsymbol{\varepsilon}$, describing $\boldsymbol{X}$ in terms of $\boldsymbol{Z}$, is fitted to obtain regression coefficients $\dot{\hat{\varphi}}_{lj} \in \dot{\hat{\boldsymbol{\Phi}}}$, and the vector of modeling errors per feature $\boldsymbol{\varepsilon} \ni \varepsilon_j$ following a multivariate normal distribution $N(0, \boldsymbol{\Sigma})$. The original model can be expressed in terms of matrix $\boldsymbol{Z}$ by substituting the coefficients of the link model $\dot{\boldsymbol{\gamma}} = \dot{\boldsymbol{\Phi}}\dot{\boldsymbol{\alpha}}$, resulting in the

bridged model $\boldsymbol{y} = \dot{\boldsymbol{Z}}\dot{\boldsymbol{\gamma}} + \boldsymbol{\epsilon}$. This comes at the cost of introducing additional modeling error $\boldsymbol{\epsilon}$ arising from the unexplained variance in the linear regression.

Adjusting the intercept in a linear model

Consider gene expression measurements $v_{ij} \in \boldsymbol{V}$ in patients indexed by $i = (1..n)$, and features indexed by $j = (1..r)$. Each feature $\boldsymbol{v_j}\ \forall \boldsymbol{j}$ is mean/variance normalized by transforming it as $\boldsymbol{x_j} = \frac{v_j - \mu_j}{\sigma_j}$ for means $\mu_j \in \boldsymbol{\mu}$ and standard deviations $\sigma_j \in \boldsymbol{\sigma}$. The result is assigned to $\boldsymbol{X}$. Consider the linear models $\boldsymbol{y} = \dot{\boldsymbol{V}}\dot{\boldsymbol{\alpha}}$ and $\boldsymbol{y} = \dot{\boldsymbol{X}}\dot{\boldsymbol{\beta}}$, that express the response vector $\boldsymbol{y}$ as a linear function of the non-mean/variance or the mean/variance normalized input respectively. Note that the weight vectors for each of the two representations can be expressed in terms of each other as $\beta_j = \alpha_j \sigma_{Vj}\ \forall j \in (1..r)$ and $\beta_0 = \alpha_0 + \sum_{\forall j} \alpha_j\ \mu_{Vj}$, or $\alpha_j = \frac{\beta_j}{\sigma_{Vj}}\ \forall j \in (1..r)$ and $\alpha_0 = \beta_0 - \sum_{\forall j} \beta_j\, \mu_{Vj}$.

References

1 Wright, V. J. et al. Diagnosis of Kawasaki Disease Using a Minimal Whole-Blood Gene Expression Signature. *JAMA pediatrics* **172**, e182293 (2018).

2 Martin, M. Cutadapt Removes Adapter Sequences from High-Throughput Sequencing Reads. *EMBnetjournal; Vol 17, No 1: Next Generation Sequencing Data AnalysisDO - 1014806/ej171200* (2011).

3 Dobin, A. et al. Star: Ultrafast Universal Rna-Seq Aligner. *Bioinformatics* **29**, 15-21 (2013).

4 Li, H. et al. The Sequence Alignment/Map Format and Samtools. *Bioinformatics* **25**, 2078-2079 (2009).

5 Lawrence, M. et al. Software for Computing and Annotating Genomic Ranges. *PLoS computational biology* **9**, e1003118 (2013).

6 Huber, W. et al. Orchestrating High-Throughput Genomic Analysis with Bioconductor. *Nature methods* **12**, 115-121 (2015).

7 Patro, R., Duggal, G., Love, M. I., Irizarry, R. A. & Kingsford, C. Salmon Provides Fast and Bias-Aware Quantification of Transcript Expression. *Nature methods* **14**, 417-419 (2017).

8 Wang, L., Wang, S. & Li, W. Rseqc: Quality Control of Rna-Seq Experiments. *Bioinformatics* **28**, 2184-2185 (2012).

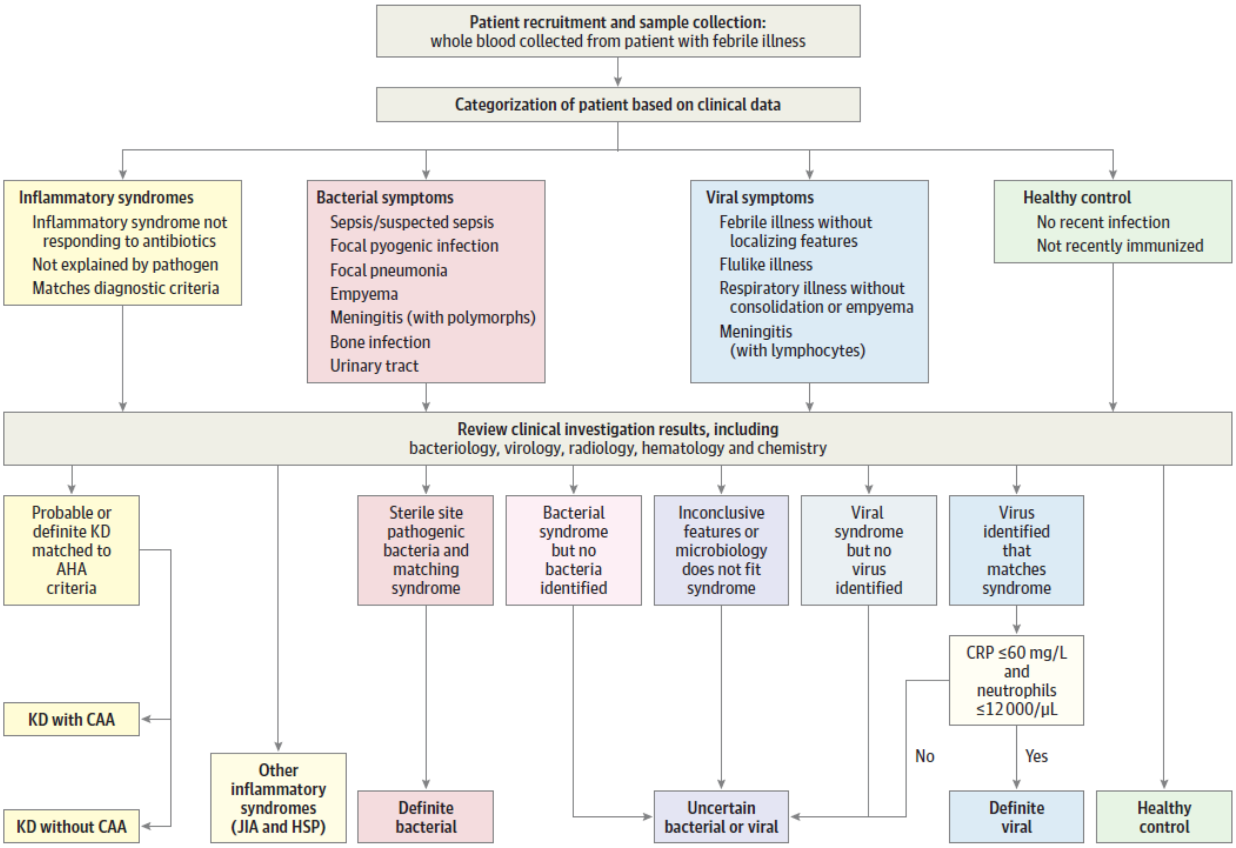

The diagnostic algorithm demonstrates the method of assigning patients to diagnostic groups. AHA indicates American Heart Association; CAA, coronary artery aneurysm; CRP, C-reactive protein; HSP, Henoch-Schönlein purpura; JIA, juvenile idiopathic arthritis; and KD, Kawasaki disease. To convert C-reactive protein level to nanomoles per liter, multiply by 9.524; to convert neutrophil count to $\times 10^9$/L, multiply by 0.001.

Figure S1. Assignment of patients to diagnostic groups as previously described.[1]

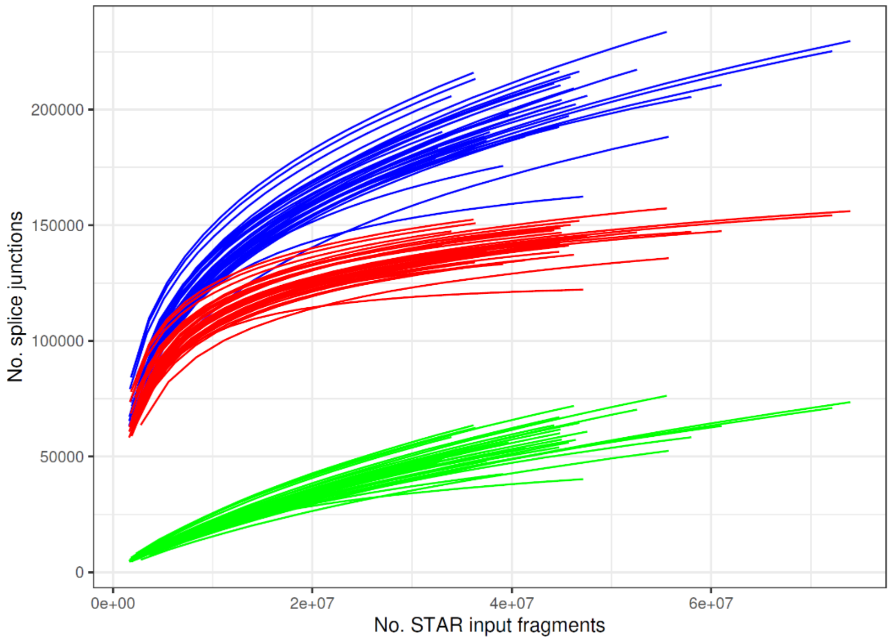

Figure S2. RNAseq splice junction saturation analysis. Shown are the number of detected splice junctions (vertical axis) at specific read depths (horizontal axis). Each line represents a sample. Colors indicate known junctions (red), novel junctions (green) and their sum (blue). The total number of known junctions in the analysis was 390,978.

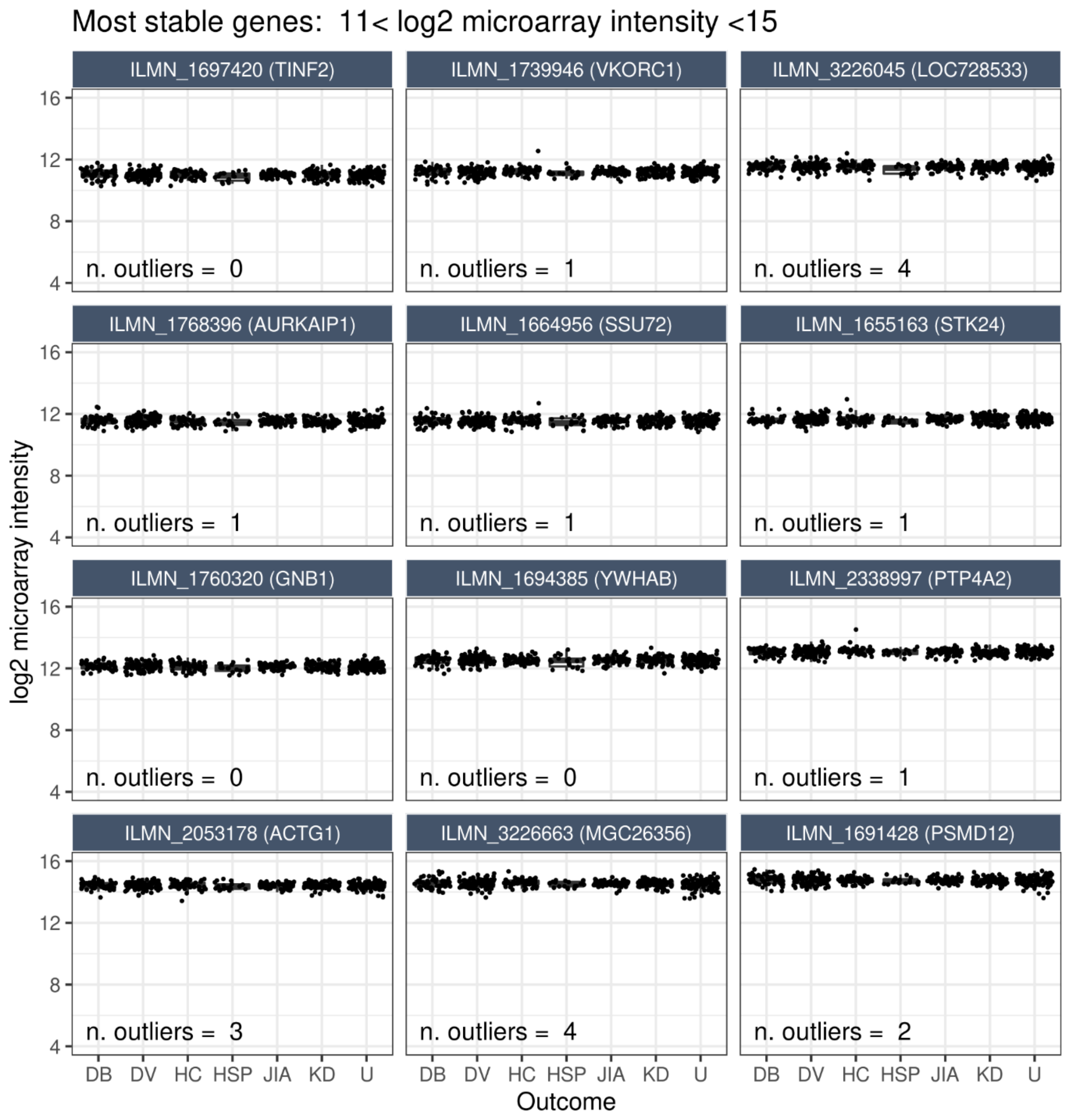

Figure S3. qRT-PCR reference gene shortlist top 12. These genes fulfilled the requirements for reference genes in the target population used for the KiDs-GEP classifier. See Table S1 for the complete short list. Each panel shows the height of expression for each of the 458 individuals per condition (KD, Kawasaki disease; DB, definite bacterial; DV, definite viral; U, infections of uncertain bacterial or viral etiology; JIA, juvenile idiopathic arthritis; HSP, Henoch-Schönlein purpura; HC, healthy control).

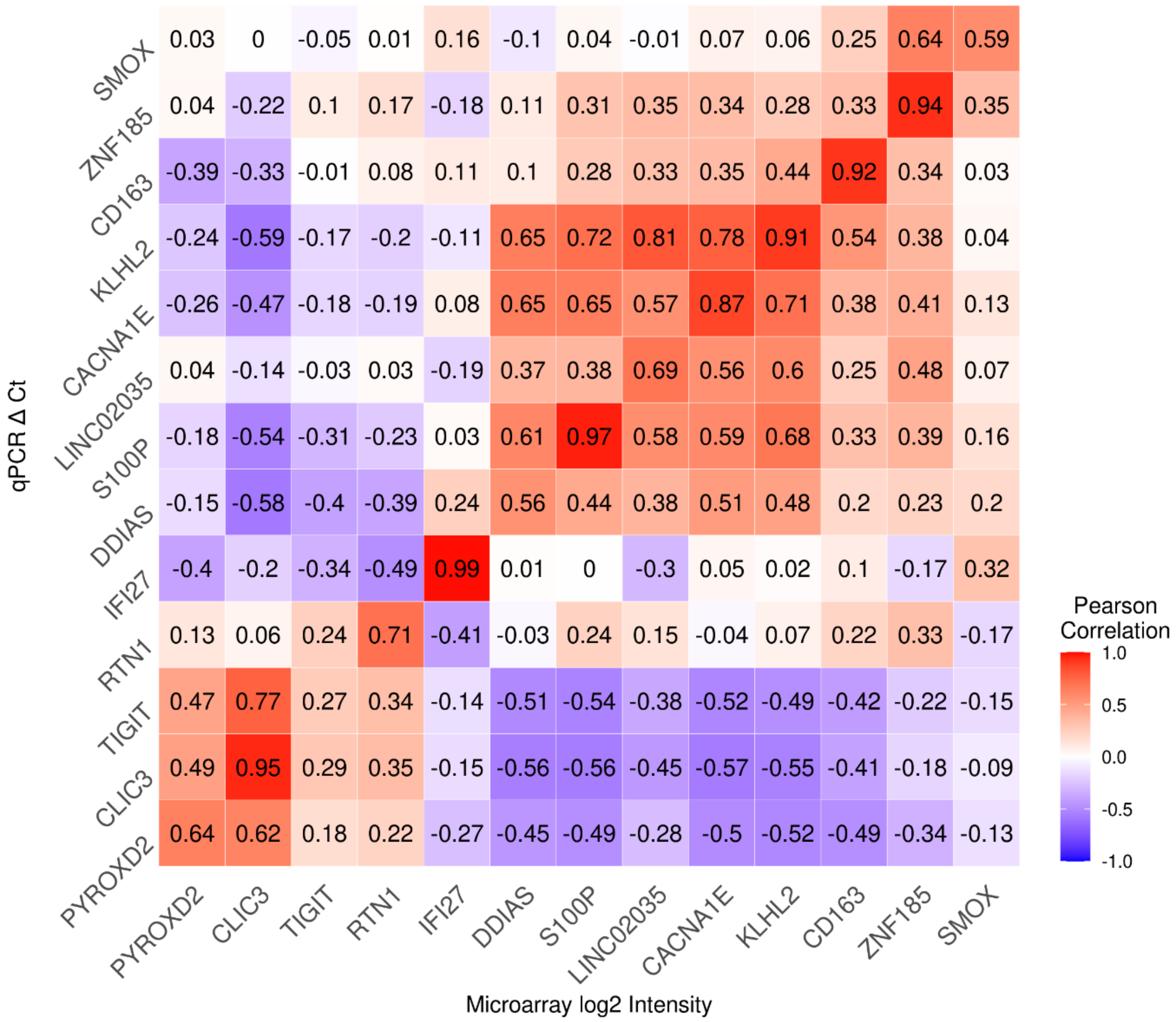

Figure S4. Correlation matrix for $\log_2$ microarray intensities versus ΔCt qRT-PCR values in the 83 samples of the bridging dataset.

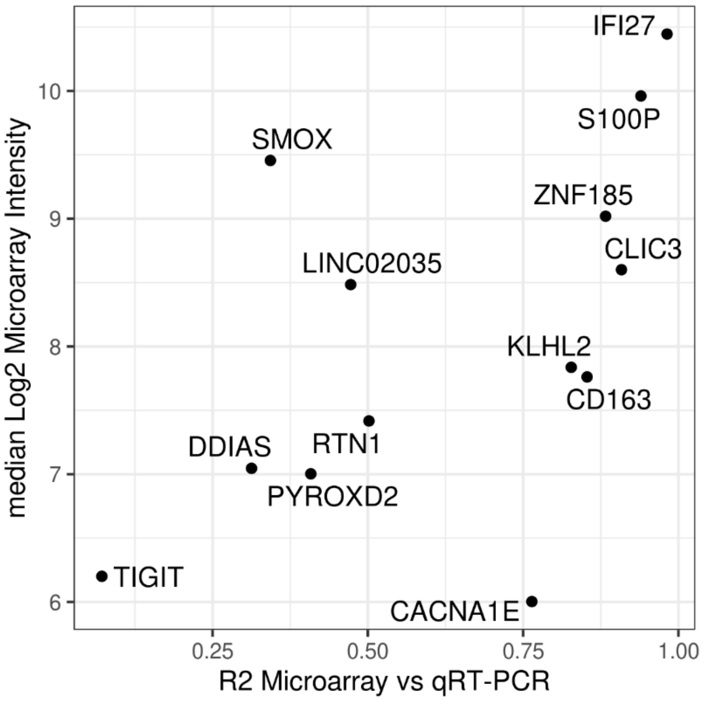

Figure S5. Association between expression level and correlation between microarray and qRT-PCR. Vertically, the median expression is shown for the 13-genes in the microarray for the 83 samples in the bridging dataset. Horizontally, the proportion (R2) of common variance between $\log_2$ microarray and ΔCt qRT-PCR values are shown.

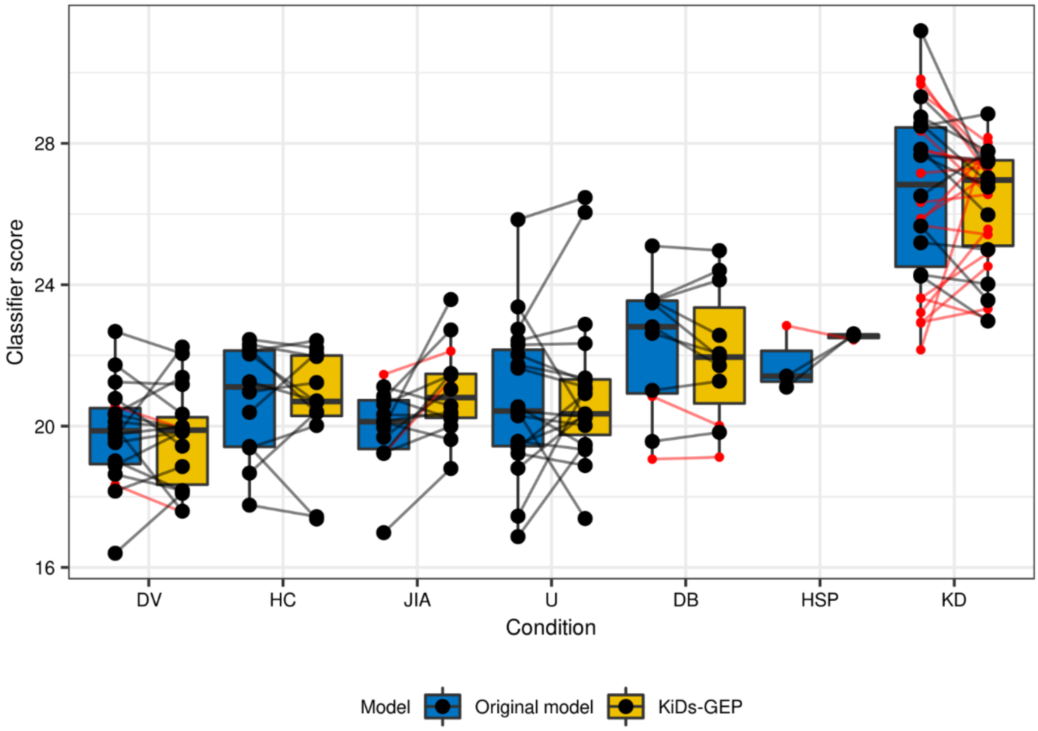

Figure S6. Pairwise classifier scores for the original model and KiDs-GEP per condition including samples with RIN≤8. Samples with RIN≤8.0 are colored in red. Paired samples are connected with a line. (KD, Kawasaki disease; DB, definite bacterial; DV, definite viral; U, infections of uncertain bacterial or viral etiology; JIA, juvenile idiopathic arthritis; HSP, Henoch-Schönlein purpura; HC, healthy control).

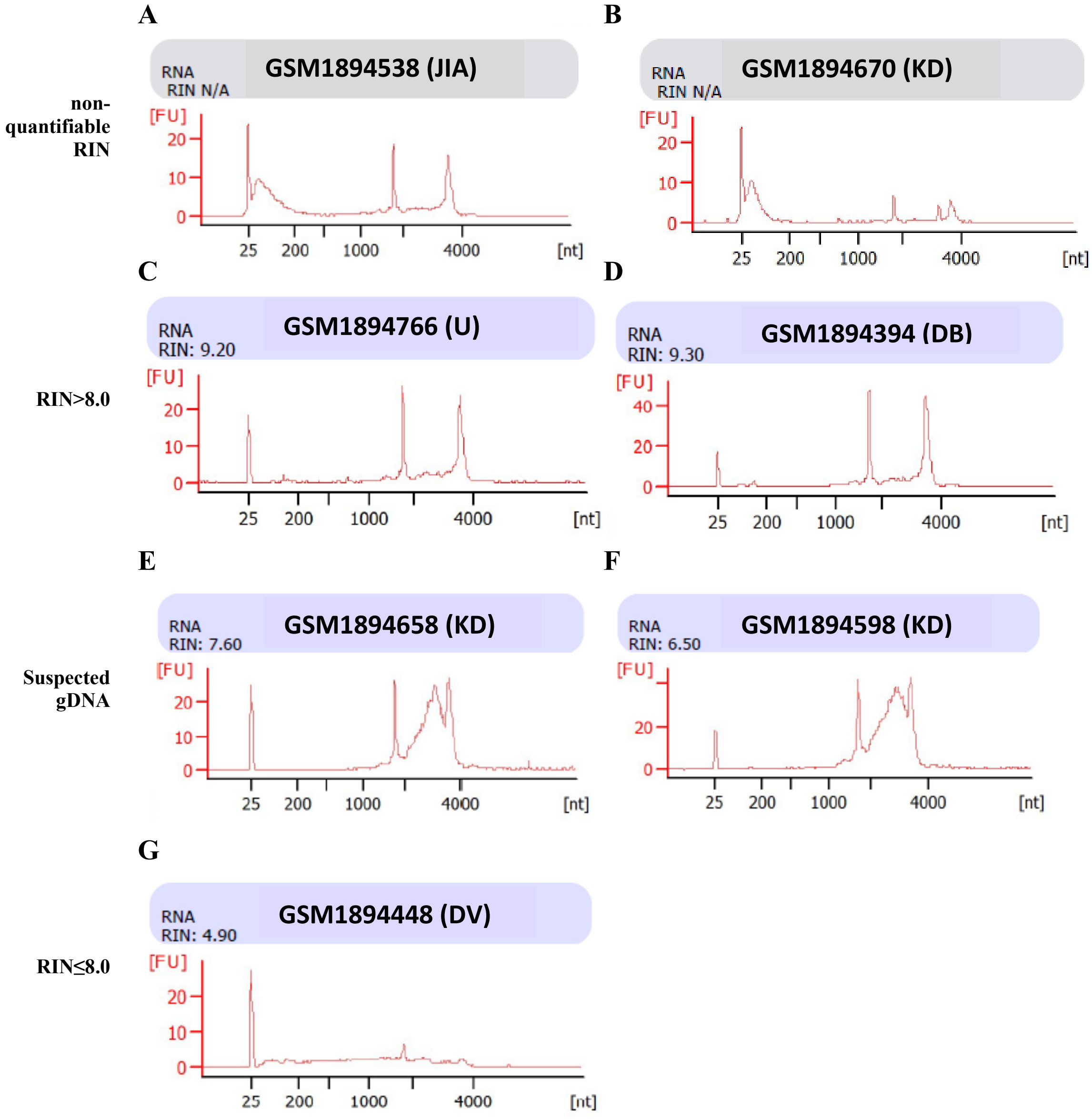

Figure S7. Electropherogram of total RNA isolated from whole blood cells and analyzed on the Bioanalyzer. Shown is a selection of samples with non-quantifiable RIN values (**A** and **B**), RIN>8 (**C** and **D**), with suspected genomic DNA (gDNA) contamination (**E** and **F**) and RIN≤8.0 (**G**). The plots show peaks for the marker (~25nt), the 18S (~1900nt), and 28S (~4000nt) ribosomal RNA (rRNA) sequences. For the samples with unknown RIN, the signal after the marker (~25nt) suggest RNA is degraded. In all samples that were off diagonal in the LINC02035 qRT-PCR vs microarray (mostly with RIN≤8), an unexpected signal, potentially gDNA, is evident between the 18S and 28S rRNA peaks. Although the RNA integrity is probably sufficient, the RIN value is likely negatively impacted by gDNA contamination.
